# Involvement of the endogenous opioid system in the psychopharmacological actions of ethanol: the role of acetaldehyde

**DOI:** 10.3389/fnbeh.2013.00093

**Published:** 2013-07-31

**Authors:** Laura Font, Miguel Á. Luján, Raúl Pastor

**Affiliations:** Area de Psicobiología, Universitat Jaume ICastellón, Spain

**Keywords:** ethanol, acetaldehyde, endogenous opioid system, salsolinol, behavior, animal

## Abstract

Significant evidence implicates the endogenous opioid system (EOS) (opioid peptides and receptors) in the mechanisms underlying the psychopharmacological effects of ethanol. Ethanol modulates opioidergic signaling and function at different levels, including biosynthesis, release, and degradation of opioid peptides, as well as binding of endogenous ligands to opioid receptors. The role of β-endorphin and µ-opioid receptors (OR) have been suggested to be of particular importance in mediating some of the behavioral effects of ethanol, including psychomotor stimulation and sensitization, consumption and conditioned place preference (CPP). Ethanol increases the release of β-endorphin from the hypothalamic arcuate nucleus (NArc), which can modulate activity of other neurotransmitter systems such as mesolimbic dopamine (DA). The precise mechanism by which ethanol induces a release of β-endorphin, thereby inducing behavioral responses, remains to be elucidated. The present review summarizes accumulative data suggesting that the first metabolite of ethanol, the psychoactive compound acetaldehyde, could participate in such mechanism. Two lines of research involving acetaldehyde are reviewed: (1) implications of the formation of acetaldehyde in brain areas such as the NArc, with high expression of ethanol metabolizing enzymes and presence of cell bodies of endorphinic neurons and (2) the formation of condensation products between DA and acetaldehyde such as salsolinol, which exerts its actions via OR.

## Ethanol and the opioid system

Evidence indicates that ethanol modulates the activity of different components of the endogenous opioid system (EOS), with a large body of data supporting the implication of opioid ligands and receptors in the mediation of some of the psychopharmacological effects of ethanol.

### The endogenous opioid system at a glance

The opioid peptide precursors proopiomelanocortin (POMC), proenkephalin (PENK) or prodynorphin (PDYN) (Kieffer and Gavériaux-Ruff, 2002) are the source for the respective peptides β-endorphin, enkephalin, and dynorphin (Nylander and Roman, 2012). These endogenous ligands activate G-protein-coupled µ-, ∂-, and κ-opioid receptors (OR) (µ-OR, ∂-OR and κ-OR), which differ in their affinities and response profiles (Evans et al., 1992; Knapp et al., 1995; Kieffer and Evans, 2009). β-endorphin presents higher affinity for µ- than ∂-, and reduced affinity for κ-OR (Roth-Deri et al., 2008; Trigo et al., 2010). Enkephalin binding to ∂-OR is greater than that for µ-OR (Khachaturian et al., 1985; Raynor et al., 1994; Akil et al., 1998) and dynorphin shows specific affinity for κ-OR (Chavkin et al., 1982; Simon, 1991; Roth-Deri et al., 2008; Trigo et al., 2010). Ethanol can modulate opioidergic transmission at different levels, including synthesis, release, and degradation of opioid peptides, and binding of endogenous ligands to OR (for a review see, Méndez and Morales-Mulia, 2008). Since β-endorphin signaling has been specially implicated in the behavioral effects of ethanol, the present review will focus on the effects of ethanol on this component of the EOS. In this regard, although OR and ligands are widely distributed through the brain, there are important neuroanatomical determinants related to β-endorphin distribution that are worth highlighting. β-endorphin-synthesizing cell bodies are primarily located in the hypothalamic arcuate nucleus (NArc) (Chronwall, 1985). Important brain regions for drug-induced effects such as the nucleus accumbens (NAcb) are under tonic control of β-endorphinic innervations from the NArc (Chronwall, 1985; Khachaturian et al., 1985; Spanagel et al., 1992; Gianoulakis, 2001). These NArc β-endorphin projections exert this control through the direct activation of OR located at the NAcb and by an indirect pathway via OR in the ventral tegmental area (VTA), which in turn modulate NAcb activity via VTA-NAcb dopamine (DA) neurons (Mansour et al., 1988; Di Chiara and North, 1992; Spanagel et al., 1992).

### Ethanol-induced modulation of β-endorphinic neurotransmission

Acute administration of ethanol induces the release of β-endorphin; an effect found in hypothalamic cell cultures and tissue preparations (Gianoulakis, 1990; Boyadjieva and Sarkar, 1994; de Waele et al., 1994; Reddy et al., 1995; De et al., 2002). Ethanol also produces *in vivo* increases in β-endorphin content at the level of the hypothalamus (Schulz et al., 1980; Patel and Pohorecky, 1989), NAcb (Anwer and Soliman, 1995; Olive et al., 2001; Marinelli et al., 2003a), midbrain including the VTA (Rasmussen et al., 1998; Jarjour et al., 2009) and the central amygdala (CeA) (Lam et al., 2008). Some studies, however, have found inconsistent results, probably related to procedural and methodological differences (Seizinger et al., 1983; Popp and Erickson, 1998; Rasmussen et al., 1998; Leriche and Méndez, 2010). Increased levels of enkephalin in the hypothalamus (Schulz et al., 1980; Seizinger et al., 1983; Milton et al., 1991) and NAcb (Marinelli et al., 2003b) have also been found after acute ethanol.

Long-term exposure to ethanol primarily induces a decrease in POMC expression (Boyadjieva and Sarkar, 1997; Rasmussen et al., 2002; Oswald and Wand, 2004) and in hypothalamic β-endorphin release and levels (Boyadjieva and Sarkar, 1994; Oswald and Wand, 2004). A limited number of studies reported an increase in biosynthesis of POMC and POMC mRNA expression (Seizinger et al., 1984; Gianoulakis et al., 1988) as well as an initial increase followed by a gradual return to normal levels (Wand, 1990). Also, some authors found an increase or no effect on β-endorphin release (Boyadjieva and Sarkar, 1994; Oswald and Wand, 2004). Discrepancies might be attributable to the method of ethanol administration, ethanol dose, time course of drug exposure, administration route and differences in the development of tolerance. Also, it has been observed that alcohol-induced changes depend on the brain region investigated as well as the species and strain of animals used (Gianoulakis, 2001; Méndez and Morales-Mulia, 2008).

### Evidence of behavioral effects of ethanol mediated by the endogenous opioid system

Given that β-endorphin, and also enkephalin, activate μ-OR, extensive research has investigated the role of μ-OR in the behavioral effects of ethanol (Gianoulakis, 1993; Herz, 1997; Sanchis-Segura et al., 2000; Thorsell, 2013). Here we will focus on the involvement of these components of the EOS in several behavioral effects of ethanol, including psychomotor stimulation and sensitization, consumption, and associative learning (with a special focus on conditioned place preference (CPP)).

#### Psychomotor stimulation and sensitization

Increased psychomotor stimulation induced by ethanol in mice can be blocked with non-selective opioid receptor antagonists such as naloxone or naltrexone (Kiianmaa et al., 1983; Camarini et al., 2000; Sanchis-Segura et al., 2004; Pastor et al., 2005; Pastor and Aragon, 2006). Some pharmacological strategies have suggested the existence of three so-called subtypes of µ-OR; µ_1_, µ_2_, and, µ_3_ (Pasternak, 2001a,b; Cadet et al., 2003) and several studies have shown that μ- and specifically the µ_1/2_ - and µ_3_-OR subtypes, but not δ- or κ-OR, are involved in the motor stimulant effects of ethanol in adult mice (Pastor et al., 2005), and also in rats during early development (Arias et al., 2010; Pautassi et al., 2012). Other studies conducted in mice have suggested that this involvement of μ-OR in ethanol stimulation is debatable (Cunningham et al., 1998; Gevaerd et al., 1999; Holstein et al., 2005). Consistent with the EOS involvement, however, a lesion of the NArc produces a decrease in ethanol-induced stimulation in mice (Sanchis-Segura et al., 2000), and knockout mice deficient in β-endorphin showed attenuated ethanol-induced stimulation (Dempsey and Grisel, 2012). Also, in rats, naltrexone prevents activation produced by ethanol when locally administered in the NArc (Pastor and Aragon, 2008) and intra-VTA blockade of the μ-OR using either naltrexone or the irreversible and selective μ-OR antagonist β-funaltrexamine reduces ethanol-induced locomotor stimulation (Sánchez-Catalán et al., 2009). Additionally, chronic naltrexone, which upregulates μ-OR (Unterwald et al., 1998; Lesscher et al., 2003), enhances the stimulant effects of ethanol in mice (Sanchis-Segura et al., 2004).

A critical role of the EOS in the motor sensitizing effects of ethanol has also been proposed (Camarini et al., 2000; Miquel et al., 2003; Pastor and Aragon, 2006). Unspecific OR antagonism prevents development (Camarini et al., 2000) but not expression (Abrahao et al., 2008) of ethanol-induced locomotor sensitization. μ-OR are particularly involved in ethanol sensitization (Camarini et al., 2000), without a clear role of any of the µ-OR subtypes in mediating this process; µ_1/2_ -OR antagonism slowed down, but did not block development of sensitization (Pastor and Aragon, 2006). Facilitation of ethanol-induced sensitization found after a period of voluntary alcohol consumption in mice was also seen to be absent in μ-OR deficient CXBK mice (Tarragón et al., 2012). The involvement of μ-OR in ethanol sensitization might be related to ethanol-induced increases in β-endorphin release as a recent study demonstrated that β-endorphin-deficient mice do not show locomotor sensitization to ethanol (Dempsey and Grisel, 2012). Also, animals with selective lesions of the NArc show prevented sensitization to ethanol (Miquel et al., 2003; Pastor et al., 2011). Altogether these data suggest that opioids and specifically β-endorphins, via μ-OR, might be critical mediators of ethanol-induced neuroplasticity underlying psychomotor sensitization.

#### Ethanol consumption

Numerous studies conducted during the last few decades showed that systemic, as well as local administration of opioid antagonists decrease ethanol consumption under a variety of schedules in different animal species (for reviews see Herz, 1997; Gianoulakis, 2001; Oswald and Wand, 2004; Modesto-Lowe and Fritz, 2005). These conclusions have also been supported by the use of OR knockout mouse models (Roberts et al., 2000; Méndez and Morales-Mulia, 2008). This strong pre-clinical basis has lead to the use of opioid antagonists in alcoholism pharmacotherapy (O’Malley et al., 1992). In rodents, the use of non-selective, as well as selective μ-OR antagonists proved to be effective at reducing ethanol consumption (Méndez and Morales-Mulia, 2008). However, the effects of these manipulations have been seen to be, in some cases, non-specific; fat, saccharin, sucrose and water intake were also reduced by these manipulations (Krishnan-Sarin et al., 1995; Nielsen et al., 2008; Rao et al., 2008; Simms et al., 2008; Corwin and Wojnicki, 2009; Wong et al., 2009). These data are compatible with the interpretation that OR, and especially μ-OR might be a key mediator of the processing of positive reinforcement, both at emotional and motivational levels (Herz, 1997; Peciña and Berridge, 2005).

In general, data obtained with κ-OR or δ-OR manipulations are less conclusive. A recent review of the literature indicates that κ-OR stimulation generally antagonizes the reinforcing effects of alcohol whereas κ-OR blockade has no consistent effect (Wee and Koob, 2010). Dynorphin/κ-OR system appears to be involved in the negative reinforcing effects of ethanol by producing an aversive effect rather than by directly modulating the rewarding mechanism of ethanol (Wee and Koob, 2010; Walker et al., 2012). However, under an alcohol dependent-state, antagonism of κ-OR results effective in decreasing ethanol voluntary consumption (Wee and Koob, 2010; Walker et al., 2012). It has been reported that blockade of δ-OR either attenuates (Lê et al., 1993; Froehlich, 1995; Krishnan-Sarin et al., 1995; June et al., 1999; Hyytiä and Kiianmaa, 2001; Ciccocioppo et al., 2002), increases (Margolis et al., 2008) or has no effect on ethanol intake (Ingman et al., 2003). These discrepancies may be related to dynamic changes in δ-OR efficacy during ethanol exposure (Margolis et al., 2008). All these data support the participation of the POMC and PENK systems in maintaining alcohol consumption (Froehlich et al., 1991; Vengeliene et al., 2008).

#### Associative learning and conditioned place preference

It has been suggested that the EOS participates in the underlying mechanisms mediating conditioned effects induced by abused drugs, including ethanol. This implication is supported by two groups of experiments. On one hand, evidence indicates that OR antagonists attenuate cue-induced reinstatement of previously extinguished responding for ethanol self-administration (Lê et al., 1999; Ciccocioppo et al., 2002, 2003; Liu and Weiss, 2002; Burattini et al., 2006; Dayas et al., 2007; Marinelli et al., 2009), which suggests a role of EOS in cue-induced incentive motivational effects influencing ethanol-seeking behavior. This interpretation is consistent with clinical data showing that opioid antagonists increase abstinence duration periods in alcohol abusers (O’Malley et al., 1992), probably by reducing cue-induced seeking behavior. On the other hand, pretreatment with opioid receptor antagonism, while not influencing the acquisition of ethanol-induced CPP, reduces the expression and facilitates the extinction of this drug-free conditioned response (Bormann and Cunningham, 1997; Middaugh and Bandy, 2000; Kuzmin et al., 2003; Pastor et al., 2011). Mice lacking μ-OR also showed attenuated ethanol CPP (Hall et al., 2001). Further studies have suggested that expression of ethanol-induced CPP depends on OR located in the VTA, CeA, as well as anterior cingulated cortex (Bechtholt and Cunningham, 2005; Bie et al., 2009; Gremel et al., 2011). Additionally, a neurotoxic lesion of the β-endorphin neurons of the NArc, showed a facilitated extinction of ethanol-induced CPP (Pastor et al., 2011). β-endorphin and μ-OR appear to be therefore critically involved in the mechanisms underlying ethanol CPP. As Cunningham and collaborators have suggested, it is possible that altered opioid signaling might in turn alter conditioned motivation that normally maintains cue-induced seeking behavior during CPP testing (Cunningham et al., 1998). It is interesting to mention that pharmacological blockade of δ-OR with naltrindole in the CeA reduces expression of CPP induced by ethanol in rats (Bie et al., 2009). Activation of κ-OR has been shown to blunt acquisition of ethanol CPP (Logrip et al., 2009). Supporting these results, κ-OR knockout mice also showed enhanced ethanol CPP (Femenía and Manzanares, 2012).

## Acetaldehyde: a psychoactive metabolite

The specific mechanism by which ethanol modulates the activity of the EOS remains to be understood. Evidence indicates that one possible mechanism might involve the role of acetaldehyde, the first metabolite of ethanol (Miquel et al., 2003; Sanchis-Segura et al., 2005b; Pastor and Aragon, 2008). Acetaldehyde is a psychoactive compound that produces behavioral and neurochemical effects suggested to mediate at least some of the effects of ethanol. Acetaldehyde is self-administered orally (Peana et al., 2010, 2012; Cacace et al., 2012) and directly into the brain (Brown et al., 1979; McBride et al., 2002; Rodd-Henricks et al., 2002; Peana et al., 2011). Its administration induces CPP (Smith et al., 1984; Quertemont and De Witte, 2001; Peana et al., 2009; Spina et al., 2010) as well as behavioral stimulation and sensitization when centrally administered (Arizzi et al., 2003; Correa et al., 2003a,b, 2009; Rodd et al., 2005; Arizzi-LaFrance et al., 2006; Sánchez-Catalán et al., 2009). The oxidation of ethanol to acetaldehyde in the brain is essentially mediated by the catalase-H_2_O_2_ system (Aragon et al., 1992a; Gill et al., 1992). Reduced brain catalase activity, which have been seen to decrease ethanol-derived central acetaldehyde formation in brain tissue preparations (Hamby-Mason et al., 1997) and in the brain of free-moving rats (Jamal et al., 2007), decreases ethanol consumption (Aragon and Amit, 1992; Koechling and Amit, 1994; Correa et al., 2004; Karahanian et al., 2011), ethanol-induced locomotor stimulation (Aragon et al., 1992b; Correa et al., 1999b, 2004; Sanchis-Segura et al., 1999a,b,c; Pastor et al., 2002; Pastor and Aragon, 2008), the anxiolityc effects of alcohol (Correa et al., 2008) and modulates ethanol-induced CPP (Font et al., 2008). Strategies aimed at increasing the production of brain acetaldehyde via an enhancement in activity of the enzymatic catalase system have also been used. These manipulations produced an increase in the motor stimulant properties of ethanol in mice (Correa et al., 1999a, 2000; Pastor et al., 2002). Other ethanol-induced effects such as taste aversion (Aragon et al., 1985) and social memory recognition have also been seen to be modulated by changes in brain catalase (Manrique et al., 2005).

Apart from brain catalase manipulation, the direct inactivation of acetaldehyde has also been shown to reduce ethanol effects, including drinking (Font et al., 2006a) and alcohol-induced relapse drinking (Orrico et al., 2013), CPP (Font et al., 2006b; Peana et al., 2008) and motor stimulation (Font et al., 2005; Martí-Prats et al., 2010; Pautassi et al., 2011).

### Acetaldehyde-induced changes in the opioidergic neurotransmission

The NArc, the main site of β-endorphin synthesis in the brain, is one of areas with the highest levels of catalase expression (Moreno et al., 1995; Zimatkin and Lindros, 1996) and lower levels of the acetaldehyde-degrading enzyme aldehyde dehydrogenase (Zimatkin et al., 1992). Therefore, it has been thus suggested that catalase-dependent formation of acetaldehyde into the NArc might mediate ethanol-induced increases in the release of β-endorphin from the NArc in turn activating OR at the level of the VTA/NAcb to stimulate behavioral and neurophysiological actions (Sanchis-Segura et al., 2005a; Pastor and Aragon, 2008). Supporting this hypothesis, several authors (Reddy and Sarkar, 1993; Pastorcic et al., 1994; Reddy et al., 1995) have demonstrated that ethanol-induced increases in hypothalamic β-endorphin release are, indeed, mediated by acetaldehyde (Reddy and Sarkar, 1993; Pastorcic et al., 1994; Reddy et al., 1995). Hypothalamic cell cultures exposed to ethanol (12.5–100 µM) led to the formation of acetaldehyde (8–24 µM) and similar concentrations of acetaldehyde (12.5–50 µM) were able to stimulate β-endorphin release when tested in the absence of ethanol (Reddy and Sarkar, 1993; Pastorcic et al., 1994). Moreover, pre-treatment of hypothalamic cell cultures with catalase inhibitors caused dose-dependent decreases in ethanol-stimulated β-endorphin secretion (Reddy et al., 1995).

Another line of research linking the EOS and acetaldehyde is the investigation of the actions of salsolinol (for a review see Hipólito et al., 2012), the condensation product of DA and acetaldehyde. Salsolinol has been shown to alter enkephalin-receptor site binding (Lucchi et al., 1982) and other OR an effect that is blocked by naloxone (Fertel et al., 1980). Interestingly, intra-NAcb administration of salsolinol increases DA levels when microinjected in the core and decreases DA levels if the administration is in the NAcb shell (Hipólito et al., 2009) in a similar way to μ- and δ-OR agonists (Hipólito et al., 2008). It has been demonstrated that μ1-OR receptors exert a tonic modulatory control over activity of the DA system (Di Chiara and North, 1992; Devine et al., 1993). Thus, one possible mechanism by which salsolinol exerts its effects on the OR could be disinhibiting DA neurons in the VTA. Upholding this hypothesis, intra-posterior VTA administration of salsolinol induces a μ-OR dependent increase in DA levels in the NAcb shell (Hipólito et al., 2011). Accordingly, it has been recently shown that salsolinol excites DA neurons of the VTA, by activating µ-OR on local GABA interneurons (Xie et al., 2012).

### Evidence of behavioral effects of acetaldehyde mediated by the endogenous opioid system

Whereas accumulating evidence indicates that the EOS participates in the behavioral effects of ethanol, only few studies have studied the involvement of this system in acetaldehyde effects. Self-administration of acetaldehyde appears to be mediated by the EOS; high doses of naloxone reduced intravenous acetaldehyde self-administration in rats, and naltrexone reduced the maintenance, the deprivation effect, and operant break points of acetaldehyde voluntary consumption (Myers et al., 1984; Peana et al., 2011). Treatment with naloxonazine, a specific µ1-OR antagonist reduces maintenance of acetaldehyde oral self-administration (Peana et al., 2011). Blockade of μ-OR using either naltrexone or the irreversible and selective μ-OR antagonist β-funaltrexamine suppress the locomotor stimulation effect of acetaldehyde when microinjected into the rat posterior VTA (Sánchez-Catalán et al., 2009). Additionally, Hipólito et al. (2010) have provided data supporting the hypothesis that acetaldehyde may mediate the actions of ethanol through a mechanism dependent on μ-OR activation. These authors showed that intra-posterior VTA injections of salsolinol induced locomotor stimulation and sensitization in rats; stimulation (but not sensitization) was prevented by μ-OR antagonism. Finally, Sanchis-Segura et al. (2005b) demonstrated that administration of a catalase inhibitor directly into the NArc is sufficient to prevent the effects of ethanol on rat locomotion. Conversely, locomotor stimulation induced by ethanol injected directly into the NArc, was prevented by catalase inhibition or naltrexone, indicating a link between the behavioral effects of a reduction in acetaldehyde formation and the antagonism of μ-OR (Pastor and Aragon, 2008). The NArc, therefore, may represent a critical site to link two independent but related hypotheses: (1) the hypothesis proposing that acetaldehyde may mediate some of the psychopharmacological actions attributed to ethanol (Aragon et al., 1992a; Smith et al., 1997; Quertemont et al., 2005; Correa et al., 2012) and (2) the hypothesis that suggests that the β-endorphin/µ-OR system participate in the reinforcing and psychomotor effects of ethanol (Stinus et al., 1980; Herz, 1997; Gianoulakis, 2001; Sanchis-Segura et al., 2005b; Pastor and Aragon, 2008). Early findings also suggested a role of the opioidergic system in mediating CPP induced by salsolinol in rats (Matsuzawa et al., 2000). Antagonism of μ-OR attenuated CPP induced by salsolinol when achieved under fear stress (Matsuzawa et al., 2000). Moreover, intra-posterior VTA administration of salsolinol, that produced CPP in rats, also produced an increase in DA in the NAcb that was suppressed by β-funaltrexamine administration (Hipólito et al., 2011).

## Summary and perspectives

In the present review we have summarized consistent results indicating that the EOS, and particularly β-endorphin and μ-OR, are critically involved in the psychopharmacological effects of ethanol. Additionally, we have reviewed a large body of data that indicates that the first metabolite of ethanol, acetaldehyde, might be responsible for the activation of β-endorphin release and μ-OR signaling after ethanol administration. There are two main lines of research suggesting a link between acetaldehyde and the EOS: (1) formation of acetaldehyde in brain areas such as the NArc, with high expression of ethanol metabolizing enzymes and presence of cell bodies of endorphinic neurons and (2) the formation of condensation products between DA and acetaldehyde such as salsolinol, which exerts its actions via μ-OR. To a certain degree both lines of research show important incompatibility. The fact that the lesions of the NArc are sufficient to block ethanol-induced behaviors challenge the putative role of salsolinol formed in other non-hypothalamic areas. Future studies will need to explore how to reconcile those two sets of data, and to clarify what is sufficient and/or necessary for acetaldehyde to induce behavioral responses mediated by the EOS. Finally, it is interesting to mention that most of the data suggesting a role of the EOS in acetaldehyde-induced behavioral effects have been linked to acetaldehyde-induced changes in the opioid system that are suggested to impact behavior via modulation of the DA system (Peana et al., 2011). Ethanol as well as acetaldehyde activate firing of dopaminergic neurons in the VTA (Foddai et al., 2004; Diana et al., 2008) and stimulate DA transmission in the NAcb (Melis et al., 2007; Enrico et al., 2009; Sirca et al., 2011), effects that are prevented by D-penicillamine, a sequestering agent of acetaldehyde (Enrico et al., 2009). A recent study demonstrates that in rats, ethanol and acetaldehyde induce via DA D1 receptors, ERK phosphorylation in the NAcb and extended amygdala (Vinci et al., 2010). This effect is blocked by D-penicillamine and by naltrexone, suggesting that the opiodergic modulation of the reinforcing properties of acetaldehyde could be mediated by the dopaminergic system (Vinci et al., 2010; Peana et al., 2011). There are other effects such as ethanol-induced CPP, ethanol drinking in some non-operant conditions and even ethanol-induced sensitization that appear to have a less straightforward involvement of DA signaling (Risinger et al., 1992; Broadbent et al., 1995; Spina et al., 2010; Young et al., 2013). Future research will need to investigate DA-dependent and independent mechanisms by which acetaldehyde might induce behavioral responses via its modulation of the EOS.

## Conflict of interest statement

The authors declare that the research was conducted in the absence of any commercial or financial relationships that could be construed as a potential conflict of interest.
